# One-Pot Synthesis of Biocompatible CdSe/CdS Quantum Dots and Their Applications as Fluorescent Biological Labels

**DOI:** 10.1007/s11671-010-9774-z

**Published:** 2010-09-17

**Authors:** Chuanxin Zhai, Hui Zhang, Ning Du, Bingdi Chen, Hai Huang, Yulian Wu, Deren Yang

**Affiliations:** 1 State Key Lab of Silicon Materials and Department of Materials Science and Engineering, Zhejiang University, 310027 Hangzhou, People's Republic of China; 2 Department of Surgery, the Second Affiliated Hospital, School of Medicine, Zhejiang University, 310027 Hangzhou, People's Republic of China

**Keywords:** Polyol Synthesis, CdSe/CdS, Quantum dots, Nanomaterials, Luminescence, Labeling

## Abstract

We developed a novel one-pot polyol approach for the synthesis of biocompatible CdSe quantum dots (QDs) using poly(acrylic acid) (PAA) as a capping ligand at 240°C. The morphological and structural characterization confirmed the formation of biocompatible and monodisperse CdSe QDs with several nanometers in size. The encapsulation of CdS thin layers on the surface of CdSe QDs (CdSe/CdS core–shell QDs) was used for passivating the defect emission (650 nm) and enhancing the fluorescent quantum yields up to 30% of band-to-band emission (530–600 nm). Moreover, the PL emission peak of CdSe/CdS core–shell QDs could be tuned from 530 to 600 nm by the size of CdSe core. The as-prepared CdSe/CdS core–shell QDs with small size, well water solubility, good monodispersity, and bright PL emission showed high performance as fluorescent cell labels in vitro. The viability of QDs-labeled 293T cells was evaluated using a 3-(4,5-dimethylthiazol)-2-diphenyltertrazolium bromide (MTT) assay. The results showed the satisfactory (>80%) biocompatibility of as-synthesized PAA-capped QDs at the Cd concentration of 15 μg/ml.

## Introduction

Fluorescent semiconductor nanocrystals, also known as one kind of quantum dots (QDs), are of considerable interest and under intensive research as biological labels either in vitro or in vivo, not only because of their bright, photostable fluorescence but also because of the broad excitation spectrum and narrow, size-controlled emission, which allows multi-color imaging [1,2]. Among them, cadmium selenide (CdSe) QDs have become one family of the most extensively studied fluorescent semiconductor nanocrystals due to their suitable and tunable band gap throughout the visible spectrum [3]. The high-temperature chemical reaction was a well-known approach for the synthesis of highly crystalline and monodisperse CdSe QDs with bright fluorescence using organometallic or chelated cadmium and phosphine-coordinated selenium as precursors [4-6]. However, besides the use of expensive, toxic chemicals, the as-received QDs were usually hydrophobic and must be converted into water-soluble nanocrystals through surface ligand exchanges [7] or encapsulations of polymers [8] and thin silica layers [9] for biological applications. The possible weight loss and decrease in quantum yields are always unavoidable during the conversion [7].

Synthesis directly in water-soluble solvent has been considered to be an alternative approach for circumventing the above-mentioned disadvantages. Recently, great efforts have been employed to focus on the synthesis of hydrophilic CdSe QDs directly in water or inverse micelles [10,11]. However, the crystal quality and quantum yields of the as-synthesized QDs were often limited, mainly due to the low reaction temperature [11]. The polyol method provided a promising high-temperature hydrophilic system for one-pot synthesis of biocompatible QDs, which combined the advantages of the two above-mentioned methods [12]. In last two decades, it has been widely applied to fabricate water-soluble particles of various materials with sub-micrometer size including metals [13], alloys [14], metal oxides [15], and metal sulfides [16]. However, obtaining biocompatible QDs with a very small size, high crystal quality, and quantum yields by polyol approach still remains a tremendous challenge [12].

Herein, we have developed a novel one-pot polyol approach for the synthesis of water-soluble CdSe and CdSe/CdS type-I core–shell QDs with several nanometers in size. The one-pot method can provide high-quality biocompatible quantum dots without using expensive phosphines and complicated surface modification, which takes the advantages of simpleness, low cost, and green precursor. Moreover, the as-received QDs show the tunable and bright PL emission with high quantum yields and high performance as fluorescent biological labels in vitro.

## Experimental Section

### Synthesis of CdSe QDs

In a typical synthesis, 1 g poly(acrylic acid) (PAA, MW = 1,800) and 0.5 mmol cadmium acetate (Cd(AC)_2_) were subsequently dissolved into 20 ml triethylene glycol (TREG), which were then heated to 200°C under Ar flow. After 30 min, the solution was cooled to room temperature, and 19 mg of Se powder was added. Finally, the mixture was heated to 240°C and kept for a certain period of time such as 1, 5, 60, and 120 min.

### Synthesis of CdSe/CdS QDs

As sulfur source, 19 mg thiourea was added into the above-mentioned CdSe precursor solution. The redundant Cd(AC)_2_ in the CdSe precursor solution was used as cadmium source. Subsequently, the mixture was heated to 160°C in 1 h. After the reaction for 2 h, the solution was quickly cooled to room temperature and precipitated by ethyl acetate. The resultant solid products were further purified by dialysis and ultrafiltration for cell imaging.

### In Vitro Cell Viability and Cell Imaging

Human embryonal kidney cell line 293T cells (ATCC CRL-11268, American Type Culture Collection, Manassas, VA) were cultured in a high-glucose Dulbecco's modified Eagle's medium (H-DMEM; Gibco, Grand Island, NY) containing 10% fetal bovine serum (FBS; Gibco) and 1% penicillin/streptomycin (Gibco) at 37°C under 5% CO_2_ condition. The cells were subcultured every 3 days. Viability of QDs-labeled 293T cells was evaluated using an MTT assay (SIGMA, St. Louis, MO). Cells were seeded in 96-well tissue culture plates at a density of 8 × 10^4^ cells/well. After 24 h, the culture medium was replaced with 200 μL of the as-synthesized QDs containing different concentrations of nanoparticles. After 24-h labeling and washing, 20 μL of a solution of MTT (5 mg/mL in PBS) was added to each well, and assay was performed at specific time intervals. The absorbance of the formazen product was then measured at a wavelength of 570 nm. Four groups of MTT tests were done for each quantum dots concentration. The values of MTT assay of labeled cells were expressed as the percentage of corresponding control cells. For cell imaging, 293T cells (2 × 10^4^ cells/24-well plates) were grown on coverslips for 24 h and then incubated with PAA-capped CdSe/CdS QDs (Cd concentration of 5 μg/mL, measured by atomic absorption spectrophotometer), at 5% CO_2_ at 37°C for 4 h. The cells were washed thrice with PBS and analyzed with confocal microscopy afterward.

### Characterization

The products were characterized by X-ray powder diffraction (XRD) using a Rigaku D/max-ga X-ray diffractometer with graphite monochromatized CuKa radiation (λ = 1.54178Å). The transmission electron microscopy (TEM) with energy-dispersive X-ray (EDX) and high-resolution transmission electron microscopy (HRTEM) was applied to determine the morphology and structure. The photoluminescence (PL) examination was performed on a detector PMT and ACTON SpectraPro 2500i using a He–Cd laser with a 325-nm wavelength as the excitation source. The confocal fluorescence images were obtained with a laser scanning confocal microscope (LEICA TCS SP2).

## Results and Discussion

**Figure 1:**
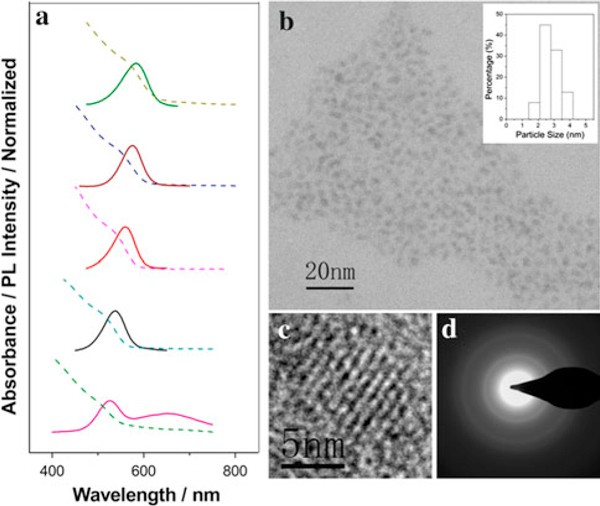
**a Temporal evolution of UV–vis absorption (*dash*) and PL (*solid*) spectra of the as-prepared PAA-capped CdSe QDs dispersed in water; b TEM and size distribution histogram; c HRTEM; and d SAED images of the as-synthesized PAA-capped CdSe nanocrystals (the absorption peak around 540 nm)**.

**Figure 2:**
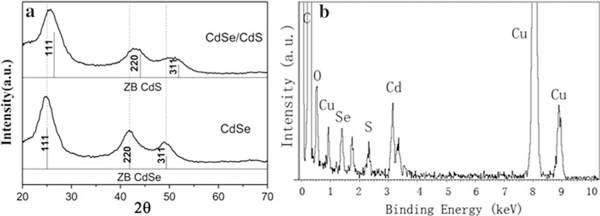
**a XRD patterns of plain CdSe and CdSe/CdS core/shell nanocrystals**. **b** EDX spectrum of the CdSe/CdS core/shell nanocrystals prepared on a copper grid.

In the present study, Cd(Ac)_2_ and Se powder are selected as source. Triethylene glycol (TREG) is used as the solvent due to its good hydrophilic feature and high boiling point (288°C). A water-soluble and biocompatible polymer with carboxylic functional groups, PAA, is selected as a capping ligand for controlling the crystal quality of QDs such as size, size distribution, and crystallinity by the formation of the chelated cadmium precursors. Moreover, since PAA is considered as a biocompatible polymer [8], we believe that the PAA could absorb on the surface of the QDs through the synthetic process, which may be advantageous for improving the hydrophilicity and biocompatibility as fluorescent biological labels. Due to the low solubility of selenium powder in TREG, no reaction had been observed at low temperature. When the temperature rose around the melting point of selenium powder (221°C), it was quickly reduced in the polyol system with reductive hydroxide groups and reacted with the carboxylate precursors forming numerous of nuclei. The explosive nucleation brings a narrow size distribution and also reduces the tendency of Ostwald ripening [17]. The nanocrystals grow larger as the extension of reaction time, causing the redshift of both absorption and emission spectra. Figure 1a shows the ultraviolet–visible (UV–vis) absorption and photoluminescence (PL) emission spectra of the PAA-capped CdSe QDs as a function of reaction time. With the extension of the reaction time, the CdSe QDs gradually grow up, and their PL emission peak can be tuned from 520 to 586 nm. The full width at half maximum (FWHM) of the PL spectra is around 50 nm. Figure 1b shows the typical TEM image of the as-synthesized PAA-capped CdSe QDs with the absorption peak around 540 nm. The average core size of as-prepared CdSe QDs calculated from the statistical results was about 2.8 nm. The HRTEM image (Figure 1c) and SAED pattern (Figure 1d) confirm the formation of the cubic CdSe QDs by PAA-assisted polyol approach. The XRD pattern of as-synthesized CdSe QDs shown in Figure 2a also shares same crystal structure with zinc-blende CdSe (JCPDS file No. 19-0191).

In the PL spectra of the CdSe QDs, there is a broad emission band originating from the surface trap sites besides the band-to-band emission, especially in the samples with smaller size, which decreases not only the monochromaticity of the fluorescence but also the quantum yields of the QDs. In our case, the quantum yields of the as-synthesized CdSe cores are around 2–3%. In order to passivate their surface trap sites and enhance the quantum yields, a consequent polyol approach was developed to fabricate type-I CdSe/CdS core–shell QDs by subsequently growing a thin CdS layer on the surface of the CdSe QDs using thiourea as sulfur source at 160°C. The XRD and EDX analysis were used to reveal the formation of CdSe/CdS core–shell QDs (Figure 2). In comparison with the XRD pattern of the CdSe QDs, the three characteristic diffraction peaks of the CdSe/CdS core–shell QDs (Figure 2a) only shift to larger angles and locate between those of the CdSe and CdS cubic phase, which demonstrate the formation of the CdS shell on the surface of the CdSe QDs [18]. The formation of the CdS shell is further supported by the EDX analysis (Figure 2b). The strong peaks for S, Se, and Cd elements in the spectrum confirm the formation of the CdSe/CdS core–shell QDs.

**Figure 3:**
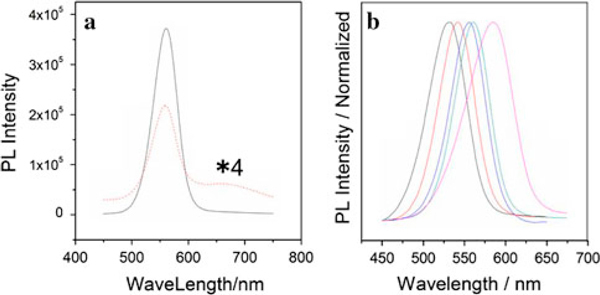
**a PL spectra of PAA-capped CdSe (*dash*) (4 time of original intensity) and CdSe/CdS (*solid*) nanocrystals**. **b** Normalized fluorescence emission spectrum of CdSe/CdS QDs with various size.

Figure 3a shows a comparison of the PL spectra of the as-prepared CdSe and CdSe/CdS core–shell QDs. As observed, the PL emission at 650 nm originating from trap sites was completely inhibited by coating a thin CdS layer on the surface of CdSe QDs due to the surface passivation [19]. Meanwhile, the brighter luminescence was achieved. Moreover, the PL emission originating from the band to band of the CdSe/CdS core–shell QDs can be tuned from 531 to 590 nm by the size of CdSe (Figure 3b) with FWHM of 40–60 nm and a quantum yield of about 30% compared with Rhodamine B [20], which has been significantly improved comparing with the CdSe cores. The PAA-capped QDs are stable for several months without precipitation in aqueous dispersion. Therefore, the PAA-capped CdSe/CdS core–shell QDs with small size, well water solubility, good monodispersity, and bright PL emission show the promising applications as fluorescent biological labels.

**Figure 4:**
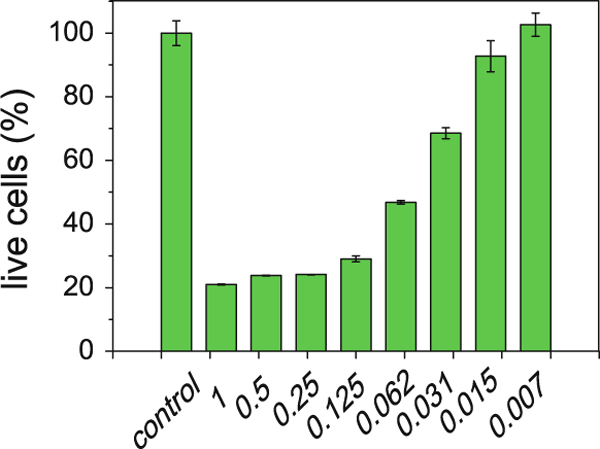
**Cell viability of Human embryonal kidney cell line 293T cells labeled with different concentration of QDs (mg Cd per mL) for 24 h at 37°C as measured by an MTT assay**. The *error bars* show the standard deviations.

Human embryonal kidney cell line is chosen as typical kind of human cells to demonstrate the promising applications as fluorescent biological labels. MTT assays were performed to evaluate the cytotoxicity corresponding to the biocompatibility of PAA-capped QDs on 293T cells. Four groups of MTT tests were done for each quantum dots concentration. In Figure 4, the cell viability shows the average cell viability of four tests, while the error bars show the standard deviations. Satisfactory (>80%) biocompatibility of as-synthesized PAA-capped QDs is achieved at a particle concentration below 15 μg Cd/mL in 293T cell lines. No statistical difference in viability is evident with PAA-QDs-labeled cells and untreated cells for 24 h at the concentration of 7 μg Cd/mL. It is well known that without proper surface modification the Cd-related quantum dots will cause severe cell damage after 24 h. MTT analysis showed that the cell viability of MCF-7 cells was below 50% after 24-h exposure to QDs (10 mg mL^-1^) capped by mercaptopropionic acid [21]. Uncapped QDs were even more toxic [22]. As a result, further surface modification processes such as PEGylation are often taken place to enhance the biocompatibility [23]. In our case, PAA absorbed on the surface improves the hydrophilicity and biocompatibility of the nanoparticles. The cytotoxicity tests indicate that, without further surface modification, the as-synthesized PAA-capped QDs show good biocompatibility as biological labels, which is comparable with PEGylated nanoparticles [23]. It turns out that the PAA modification during the "one-pot" synthesis is both simple and effective.

**Figure 5:**
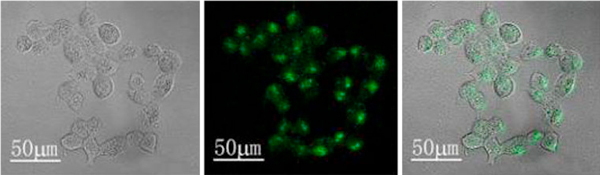
**Confocal microscopic visualization of Human embryonal kidney cell line 293T cells treated with PAA-capped green-emitting CdSe/CdS QDs (λ_em max_ = 559 nm, about 3 nm in size) with the concentration of 5 μg/mL for 4 h at 37°C**. From *left* to *right*, the panels show the transmission image, luminescence image, and an overlay of the two.

For their in vitro cell labeling studies, the cultured human embryonal kidney cell line 293T cells were incubated with the PAA-capped CdSe/CdS core–shell QDs (λ_em max_ = 559 nm, about 3 nm in size) with the concentration of 5 μg/mL for 4 h at 37°C. After 4 h, the cells were washed thrice with PBS to remove extra nanoparticles that were not uptaken by the cells and imaged using a laser scanning confocal microscope. Figure 5 shows the typical labeling images of 293T cells with PAA-capped CdSe/CdS core–shell QDs. From these images, the bright green optical signal can be clearly observed from the cell interior. The result demonstrated that the as-synthesized quantum dots can be quickly uptaken by the 293T cells within 4 h. Moreover, we did not observe any signs of morphological damage to the cells after the treatment with PAA-capped CdSe/CdS core–shell QDs. This preliminary result indicates that the as-prepared QDs had promising applications as fluorescent biological labels.

## Conclusions

In summary, we have developed a novel, cost-effective, and environment friendly polyol approach for the one-pot synthesis of biocompatible CdSe and CdSe/CdS core–shell QDs with several nanometers in size, good biocompatibility, good monodispersity, strong, and tunable fluorescent emission. The as-synthesized PAA-capped CdSe/CdS core–shell QDs exhibited high performance as fluorescent cell labels in vitro and thus promising applications.
